# Insecticide application timing effects on alfalfa insect communities

**DOI:** 10.1093/jee/toad071

**Published:** 2023-04-21

**Authors:** Micah McClure, Judith Herreid, Randa Jabbour

**Affiliations:** Department of Plant Sciences, University of Wyoming, #3354, 1000 E University Avenue, Laramie, WY 82071, USA; Department of Plant Sciences, University of Wyoming, #3354, 1000 E University Avenue, Laramie, WY 82071, USA; Department of Plant Sciences, University of Wyoming, #3354, 1000 E University Avenue, Laramie, WY 82071, USA

**Keywords:** alfalfa weevil, lambda-cyhalothrin, spray timing, pyrethroid, generalist predators

## Abstract

Timing of insecticide application can impact efficacy, given variation in both weather and development of the crop and its insect pests. Both target and nontarget insects may vary in life stage and abundance at the time of application. In alfalfa *Medicago sativa* L. cropping systems, producers have interest in early-season insecticide applications to eliminate last-minute decisions about preharvest applications for alfalfa weevil *Hypera postica* (Gyllenhal) (Coleoptera: Curculionidae). The standard recommendation is based on scouting larvae close to the first harvest time. We compared early and standard timing of application of a lambda-cyhalothrin pyrethroid on alfalfa pest and beneficial insects. Field trials at a university research farm were conducted in 2020 and 2021. In 2020, early application was as effective as the standard timing against alfalfa weevil, as compared to the untreated control, but less effective than the standard timing in 2021. Effects of timing against *Lygus* bugs (Hemiptera: Miridae), grasshoppers (Orthoptera: Acrididae), and aphids (Hemiptera: Aphididae) were inconsistent between years. We observed the potential for early application to reduce negative impacts on ladybird beetles (Coleoptera: Coccinellidae) and spiders (Araneae), however, damsel bugs (Hemiptera: Nabidae) were similarly reduced by insecticide application regardless of timing. Overall arthropod community composition differed by both year and treatment. Future research should explore potential trade-offs of spray timing at larger spatial scales.

## Introduction

Alfalfa *Medicago sativa* L. is an economically and ecologically important crop plant commonly grown worldwide. It hosts a diversity of pest and beneficial insects that can be impacted by management decisions. Alfalfa producers in the Western United States currently face the challenge of managing insect pests effectively in the face of insecticide resistance (Rodbell et al. 2022) and potential nontarget effects of insecticide use. However, growers surveyed in Wyoming revealed insecticide use is the most common form of management for control of insect pests in alfalfa (Jabbour and Noy 2017) in contrast to biological, physical, and cultural methods. A need exists to provide information on how insecticides can be used to be both effective against pests while limiting negative impact on beneficial insects (Ternest et al. 2020, Knapp et al. 2022).

The most problematic insect pest in our region is alfalfa weevil *Hypera postica* (Gyllenhal) (Coleoptera: Curculionidae). Current recommendations for chemical management of alfalfa weevil include scouting to establish how field populations compare to economic thresholds for alfalfa weevil larvae (Harrington et al. 2021). However, the turnaround time between scouting for larvae, application, and subsequent harvest can be quite quick. When combined with unpredictable weather, coordination with outside applicators, and preharvest interval requirements, stress and risk accumulates such that producers are interested in making insecticide application decisions earlier in the season. Early application in this system in our region shows promise. Field trials in neighboring Colorado (Peairs et al. 2019) included spray timings that occurred in early spring – earlier than when scouting for alfalfa weevil larvae would be possible. These trials emerged as a result of early-season insecticide applications targeting army cutworm *Euxoa auxiliaris* (Grote, 1873) (Lepidoptera: Noctuidae) that also yielded observations of reduced alfalfa weevil densities later in the growing season in these locations. In addition, early-season applications are of interest to producers whose fields are receiving herbicide applications. Applicators offer to mix insecticide into early-season herbicide applications in alfalfa with the goal of alfalfa weevil control downstream; however, there is limited knowledge on how such mixtures could impact efficacy of insect or weed control.

The development and growth of the alfalfa weevil are heavily dependent on temperature. The lower developmental threshold for alfalfa weevil growth is 8.9°C. With this in mind, alfalfa weevil oviposition can occur before those lower thresholds, but ultimately varies among location. In the Northern United States, adults typically lay their eggs in the spring. In the southern U.S., alfalfa weevil may overwinter as eggs (Pellissier et al. 2017). Understanding how alfalfa weevil stage and insecticide application timing align can help managers decide on insecticide application logistics. In addition, preharvest and pregrazing intervals indicated on the product label contribute to this decision making. Finally, residual activity and subsequent impact on both pest and beneficial insects varies according to active ingredient (Roubos et al. 2014) and other variables such as temperature, precipitation, cloud cover, and plant biomass (Lyons and Hageman 2021).

Alfalfa, a perennial nitrogen-fixing plant, offers a hospitable environment for a diversity of arthropods (reviewed in González del Portillo et al. 2022). Beyond alfalfa weevil, pests of importance in our region include aphids (Hemiptera: Aphididae), grasshoppers (Orthoptera: Acrididae), and *Lygus* bugs (Hemiptera: Miridae). Aphid thresholds vary according to the species, and aphid density can also promote abundance of generalist predators or support parasitoid wasps that provision aphid honeydew (Elliott et al. 2002, Rand and Lundgren 2019). In Wyoming, grasshoppers can be occasionally problematic (Jabbour and Noy 2017). *Lygus* bugs are primarily insect pests of alfalfa seed production due to their preferential feeding on flowers and young seeds but occur in forage systems (Jabbour and Noy 2020). Lastly, clover root curculio *Sitona hispidulus* (Fab.) (Coleoptera: Curculionidae) is an emerging pest of alfalfa in our region. Larvae feed on the root systems, however, there is no current information on the relationship of clover root curculio densities and root damage and economic thresholds (Rim et al. 2019).

Alfalfa has documented potential to aid in conservation in agricultural landscapes (as reviewed by González del Portillo et al. 2022), including beneficial insects that can contribute to biological pest control and pollination. Common generalist predators in alfalfa include ladybird beetles (Coleoptera: Coccinellidae), damsel bugs (Hemiptera: Nabidae), and spiders (Araneae). Although ladybird beetles and damsel bug densities were associated with alfalfa weevil densities across field sites in Montana, experimental predator exclusion had no effect on alfalfa weevil densities (Rand 2017). In a different Montana field study, no spatial synchrony was found between alfalfa weevil and generalist predators (Shrestha et al. 2021). However, pea aphids were higher within exclosures, demonstrating significant pressure on pea aphids by generalist predators. Predators also contribute to *Lygus* bug predation, but generally will feed on pea aphids if available (Ouayogode and Davis 1981). When aphid abundance is very low, ladybird beetles can feed on alfalfa weevil larvae, however, it is not as beneficial to their growth and reproduction – they still require aphids as part of their diet (Kalaskar and Evans 2001). In general, generalist predators are important to alfalfa pest control, and they may be selective to pea aphids if abundance is high.

Wasps provide pest control services in a variety of agricultural systems (Wang et al. 2019, Brock et al. 2021). Predatory aculeate wasps have been shown to decrease insect pest damage and control insect pest populations (Bommarco et al. 2011, Southon et al. 2019). In alfalfa, parasitoids are important natural enemies for pests, including weevils, aphids, and *Lygus* bugs. Wasp species such as *Bathyplectes curculionis* (Thomson) (Hymenoptera: Ichneumonidae) and *Oomyzus incertus* (Ratzeburg) (Hymenoptera: Eulophidae) persist in the intermountain west from previous releases by the United States Department of Agriculture as part of an extensive classical biological control effort targeting alfalfa weevil (Kingsley et al. 1993). In addition, many parasitoid wasp families were documented in alfalfa at our research farm that could impact a variety of herbivores (Pellissier and Jabbour 2018).

In Europe, alfalfa is shown to be fairly evenly used by honey bees, bumble bees, and other wild bees (Rollin et al. 2013). In the Northern Great Plains, similar land uses and conservation efforts were found to benefit both honey bees and wild bees (Evans et al. 2018). These researchers considered alfalfa as a “bee forage” crop given its floral resources. Therefore, insecticide applications in alfalfa must also consider potential impacts on both native and honey bees.

In our study, we use a pyrethroid insecticide, nonsystemic and broad spectrum, commonly used in alfalfa. In alfalfa pest management field tests in Kansas, the pyrethroid used resulted in good control of alfalfa weevil, but also resulted in reductions of ladybird beetles and parasitoids compared to the untreated control (Michaud and Bain 2016). The indoxacarb tested in this trial was the only treatment to not reduce ladybird beetles. Pyrethroid insecticides have also been shown to be highly toxic against generalist predators species of *Geocoris* and *Orius* (Hemiptera: Anthocoridae) (Prabhaker et al. 2017). Beyond this, pyrethroids also have various sublethal effects on beneficial insect physiology and behavior (Desneux et al. 2007).

Our overall goal was to compare the effect of 2 different spray timings of lambda-cyhalothrin pyrethroid on insect communities in alfalfa. Our objectives were to examine (i) efficacy against pests of importance, (ii) nontarget effects on beneficial insects, and lastly, (iii) compare insect communities as a whole between treatments. Our hypotheses were as follows: (i) early spray timing is less effective against alfalfa weevil, (ii) early spray timing has less nontarget effects on beneficial arthropods, and (iii) arthropod communities will holistically differ between each spray timing treatment and the control.

## Materials and Methods

### Study Site

This experiment was conducted at the University of Wyoming James C. Hageman Sustainable Agricultural Research and Extension Center in Lingle, WY (42.1294°N, 104.3902°W). Glyphosate-tolerant alfalfa variety Ameristand 455TQRR was planted in an irrigated 0.81 ha block in Fall 2018 and managed according to production norms with standard harvests in the years before the experiment. No insecticide was applied to the alfalfa crop before the experiment.

### Experimental Design

The experiment had 3 treatments: (i) early spray timing, (ii) standard spray timing, and (iii) control with no insecticide application. Within the 0.81 ha field, we flagged 15 experimental plots (5 replications per treatment), embedded within continuously planted alfalfa (Supplementary Fig. 1). Plots measured 9 × 18 m with 9 × 18 m untreated buffer zones of alfalfa in between each experimental plot. Treatments were randomly assigned to each replicate to create a completely randomized designed study. The entire experiment was conducted in 2020 and 2021. In 2021, to prevent any legacy effects from the previous year’s experimental treatments, the 2021 plots were shifted laterally by 9 meters into the location of the buffer zones in 2020; therefore, the 2021 plots had no history of insecticide application.

### Experimental Treatments

Spray timings were based on label guidelines in regards to alfalfa stage or height and required preharvest intervals. In addition, we consulted with local applicators to ensure our timings matched local production practices. The early spray timing was based on alfalfa plant stage to mimic the timing used for early-season herbicide applications in alfalfa. It occurred in early May of each year (Table 1). The standard spray timing was applied when late-instar alfalfa weevils were present and with enough time to meet required preharvest intervals, in late May of each year (Table 1). Both of these applications occurred before the first cutting of the season. We used the recommended application rate for lambda-cyhalothrin (Syngenta Warrior II) at 0.14 liter/ha. All applications were conducted at 10 am for both years using a hand-held boom. Conditions for all spray dates are reported in Table 1. In both years, the early and standard spray timings were separated by 3 wk.

**Table 1.** Field conditions at spray timing in 2020 and 2021

Spray Date	May 6 2020	May 27 2020	May 4 2021	May 25 2021
Treatment	Early	Standard	Early	Standard
Plant height^a^ (cm)	22	43	19	41
Air temperature	15.1°C	19.4°C	10.6°C	22.5°C
Average wind speed (kph)	7.24	1.30	2.44	2.59
Relative humidity	0.57	0.55	0.73	0.51
Cloud cover (%)	75	10	70	0
Growing degree days^b^	50	255	65	279

^a^Average of measurements collected from 2 locations per plot.

^b^Estimate according to Harcourt 1981 model with March 1 start.

### Arthropod Communities at Time of Treatment

Arthropod communities at the time of treatment at the experimental site were characterized by sampling from the untreated alfalfa buffer zones in between plots without entering treated plots (Supplementary Fig. 1). We sampled these areas to avoid entering treated plots and avoid sampling control plots more intensively than treated plots. We stratified this sampling across the experimental site to be representative. Collections were done using a sweep net, each sample comprised of 20 sweeps. In 2020, we collected 4 samples from the buffer zones at the experimental site at each treatment date. In 2021, we increased to 6 buffer zone samples at each treatment date. Contents of the net were emptied into a labeled gallon sized plastic bag. A paper towel was placed in the bag to help absorb moisture and maintain sample quality. The bags were placed in a cooler with ice packs, transported back to the laboratory, and frozen until sorting.

### Arthropod Communities Following Treatment

One and 2 wk after the standard spray application, arthropods were collected using sweep sampling from all experimental plots, with the second week of sampling occurring 1 wk before the first alfalfa harvest from all replicates. Twenty 180-degree sweeps were conducted walking in a Z pattern within the plot, avoiding the outer 1.5 m of the plot. Contents of the samples were handled as described previously.

### Arthropod Identification

Samples were sorted to identify and count pest and beneficial arthropods of importance in alfalfa. Pest insects in the following categories were identified and counted: alfalfa weevil, *Lygus* bugs, aphids, grasshoppers, and clover root curculio. Although *Lygus* bugs are generally considered to be a pest of importance in seed alfalfa, not forage, we included it given the potential for *Lygus* bugs to be problematic in adjacent crops depending on the landscape context (Carrière et al. 2012). In other words, forage alfalfa growers also growing sunflowers or dry beans may be interested in effect of spray timing on *Lygus.* Beneficial arthropods in the following categories were identified and counted: wasps (all Hymenoptera excluding Formicidae and Anthophila), bees (Hymenoptera: Anthophila), ladybird beetles (Coleoptera: Coccinellidae), damsel bugs (Hemiptera: Nabidae), and spiders (Araneae).

### Data Analysis

Insect populations at the time of early and standard application were compared using descriptive statistics and *t*-tests. We used *t*-tests to compare early and standard timing within each experimental year. To test for differences in efficacy against pests of importance and nontarget effects on beneficial insects, we used two-way ANOVA with treatment and sample date as factors. We conducted pairwise means comparisons to determine treatment differences. We analyzed each experimental year separately given differences in stand age. Data were log-transformed or square-root transformed as needed to meet assumptions of normality. We used R project in RStudio for all data analysis (R Core Team 2022, RStudio Team 2022), including the packages “*multcomp*” (Hothorn et al. 2008) to provide multiple comparisons by adjusting *P*-values and “*emmeans*” (Lenth 2021) to separate estimated means given a fitted model. We explored the effect of spray timing on the following pests: alfalfa weevil, aphids, grasshoppers, and *Lygus* bugs. We measured the effect of spray timing on the following beneficial insect groups in alfalfa: ladybird beetles, damsel bugs, wasps, and spiders.

All arthropod counts were log transformed for multivariate analysis. A PERMANOVA was run using the package “*vegan*” (Oksanen et al. 2022) to examine the effect of year, treatment, and the interaction between year and treatment on arthropod community composition. Community composition differences were visualized using a principal components analysis.

## Results

### Insect Communities at Time of Application

Insects were less abundant at the time of the early spray application than at the time of the standard spray application in both years of the experiment. In particular, alfalfa weevil larvae were significantly less abundant during the early spray application than the standard application in both years (Table 2). Significant differences of grasshoppers and ladybird beetles were found in 2020 but not in 2021, when their populations were similarly low on both spray dates (Table 2). Aphids were significantly higher on the standard application date than the early date in 2021. In 2020, they were more abundant on the standard application date but the difference was nonsignificant given high variability (Table 2).

**Table 2.** Insect abundances at time of insecticide application for early (May 6, 2020 and May 4, 2021) and standard (May 27, 2020 and May 25, 2021) experimental treatments. Insects were collected by sweep-net from buffers in between experimental plots to represent insect populations at the experimental site. Abundances are average (S.E.) per 20 sweeps. *P*-values reported from *t*-tests comparing between application dates within each year

	6 May 2020	27 May 2020	*P*-value	4 May 2021	25 May 2021	*P*-value
Alfalfa weevil (adults)	4.75 (1.55)	8.5 (0.65)	^a^	2 (0.45)	2.5 (0.72)	
Alfalfa weevil (larvae)	0.5 (0.29)	375.75 (31.63)	^c^	0 (0)	61.33 (9.85)	^d^
Clover root curculio	9 (4.08)	21 (10.46)		0 (0)	0 (0)	
Grasshoppers	2 (1.35)	9.75 (1.89)	^b^	0 (0)	0.5 (0.34)	
Lygus bugs	2.5 (0.87)	14.75 (4.57)	^a^	1.5 (0.81)	1 (0.52)	
Aphids	7.25 (0.63)	190.75 (63.40)	^a^	5.67 (1.87)	197.67 (21.90)	^d^
Bees	0 (0)	1.25 (0.75)		0 (0)	0 (0)	
Ladybird beetles	0.75 (0.48)	4.5 (0.65)	^c^	0 (0)	0.17 (0.17)	
Damsel bugs	1.5 (0.96)	5.25 (1.49)	^a^	1 (0.26)	1 (0.37)	
Wasps	0.25 (0.25)	4.75 (1.89)	^a^	1.67 (0.33)	6.5 (1.89)	^a^
Spiders	2.25 (0.25)	2.75 (0.63)		0.33 (0.21)	1 (0.45)	

^a^ *P* < 0.1.

^b^ *P* < 0.05.

^c^ *P* < 0.01.

^d^ *P* < 0.001.

### Efficacy Against Pests of Importance

Early and standard applications reduced alfalfa weevil larval populations compared to the untreated control in both years of the experiment (Table 3). In 2020, alfalfa weevil larval abundance was highest in the untreated control but did not differ significantly between early and standard applications (*F*_2,24_ = 4.28, *P* = 0.025; Table 3). In 2021, alfalfa weevil larval abundance was highest in the control, followed by the early application, then the standard application (*F*_2,24_ = 70.53, *P* < 0.001; Table 3).

**Table 3.** Arthropod abundance, mean (S.E.), in early application, standard application, and control treatments per 20 sweeps. These collections occurred 2 wk after the standard application date in each year, with samples collected on June 11, 2020 and June 10, 2021

	2020			2021		
	Early	Standard	Control	Early	Standard	Control
Alfalfa weevil (larvae)	152.2 (25.1)	199.0 (39.9)	507.2 (164.0)	584.4 (42.0)	398.8 (53.7)	1303.6 (114)
Aphids	523.8 (132.7)	570.0 (105.2)	67.8 (15.1)	2852 (355.1)	2110.4 (459.4)	2238.4 (353.2)
Grasshoppers	57.2 (10.5)	7.6 (1.4)	36.2 (5.9)	1.6 (0.4)	0.8 (0.3)	7.6 (6.6)
Lygus bugs	56.6 (4.8)	6.6 (2.2)	103.6 (25)	3.8 (0.8)	3.8 (2.4)	173.4 (47.5)
Damsel bugs	3.4 (1.2)	0.2 (0.2)	12.2 (5.1)	3.0 (1.2)	0.6 (0.4)	36.4 (6.1)
Wasps	13.8 (2.5)	10.6 (2.0)	6.4 (1.2)	18.0 (3.7)	14.0 (2.1)	12.6 (4.4)
Ladybird beetles	30.4 (6.5)	10.2 (1.2)	12.2 (2.0)	11.4 (0.9)	4.2 (0.8)	20.6 (4.7)
Spiders	4.6 (0.9)	1.0 (0.6)	5.2 (1.6)	3.8 (1.1)	6.2 (2.1)	1.8 (0.6)
Bees	3.0 (0.8)	2.2 (1.2)	0 (0)	0 (0)	0 (0)	0 (0)

The response of aphids to these treatments varied between experimental years. In 2020, aphids were significantly higher in treated plots than in the untreated control, regardless of standard or early timing (*F*_2,24_ = 9.34, *P* < 0.001; Table 3). In 2021, aphids were higher in the untreated control 1 wk after the standard application than in both the early and standard application (Supplementary Table 1); however, 2 wk after the standard application, aphid density did not differ between any of the treatments and the control (treatment*date, *F*_2,24_ = 15.89, *P* <0.001; Table 3). Two weeks after standard application, aphid densities were more abundant at the experimental site in 2021 compared to aphid densities in 2020 (Table 3).

Grasshopper densities were significantly lower in the standard application as compared to both the early application and control treatments in 2020 (Table 3). In 2021, grasshopper densities were very low in all replicates (0–3 individuals) with one exception (34 grasshoppers swept in one control plot on June 10, 2021). Therefore, we were unable to complete a meaningful analysis of treatment effects on grasshoppers in 2021.

Both early and standard insecticide applications reduced *Lygus* bug densities compared to the untreated control in both years of the experiment (Table 3). In 2020, *Lygus* were less abundant in the standard application as compared to the early application (*F*_2,24_ = 67.49, *P* < 0.001). In 2021, treatment and sampling date interacted significantly such that *Lygus* densities were lower in the standard application than the early application 1 wk after the standard application occurred; however, densities were similar between these treatments – and both significantly lower than the control – 2 wk after the standard application (*F*_2,24_ = 6.57, *P* = 0.005).

### Nontarget Effects on Beneficial Arthropods

Ladybird beetles were less abundant in the standard application than both the early application and untreated control in the second week of sampling following standard application (Table 3). This difference was only statistically significant in 2021 (2020: *F*_2,24_ = 2.77, *P* = 0.083; 2021: *F*_2,24_ = 6.32, *P* = 0.006). In contrast, damsel bugs were less abundant in both standard and early applications as compared to the untreated controls in both years of the experiment (2020: *F*_2,24_ = 26.27, *P* < 0.001; 2021: *F*_2,24_ = 55.8, *P* < 0.001). Hymenopteran wasp density did not vary significantly according to experimental treatment in either year of the experiment. Finally, in 2020, spiders were less abundant in standard application plots than early application plots, both less abundant than the untreated controls (*F*_2,24_ = 6.12, *P* = 0.007). In 2021, although spiders were less abundant in treated plots 1 wk after standard application (Supplementary Table 1), there were no significant differences in spider abundance 2 wk after standard application (treatment*date, *F*_2,24_ = 4.4, *P* = 0.024, Table 3).

### Arthropod Communities Compared Between Treatments and Years

**Fig. 1.**
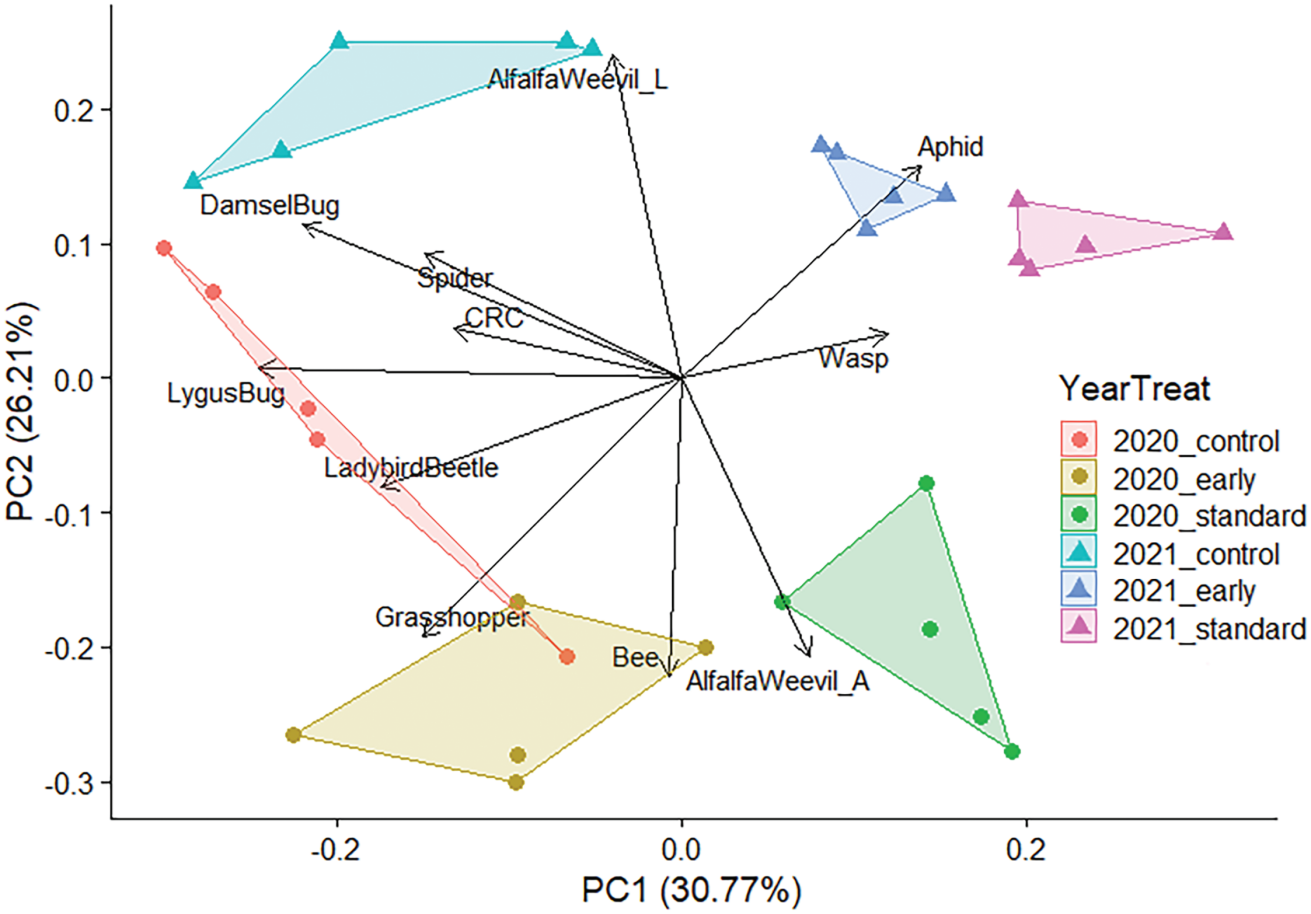
Principal component analysis biplot with principal component 1 and 2. Samples collected in 2020 are denoted with circles and 2021 samples are denoted with triangles. Color distinguishes timing treatments for each year. Arrows denote loadings for alfalfa weevil larvae (AlfalfaWeevil_L), alfalfa weevil adults (AlfalfaWeevil_A), clover root curculio (CRC), and 8 other groups of arthropods represented by their common names.

Multivariate analyses were used to compare arthropod communities between experimental treatments and years. Arthropod communities differed significantly by year (*F*_1,24_ = 32.5, *P* = 0.001), treatment (*F*_2,24_ = 19.8, *P* = 0.001), and an interaction between treatment and year (*F*_2,24_ = 3.5, *P* = 0.004). The principal component analysis illustrates these distinct clusters for each year*treatment (Fig. 1). The first principal component explained 30.77% of variation in community composition, with the highest loadings from *Lygus* bugs, damsel bugs, and ladybird beetles. All 3 of these taxa were more abundant in the experimental controls of 2020 and 2021, along with the 2020 early application treatment. The second principal component explained an additional 26.21% of variation in community composition, with the highest loadings from alfalfa weevil larvae, bees, and alfalfa weevil adults. Abundance of alfalfa weevil larvae was associated with the 2021 experimental year given higher densities that year. Bees and alfalfa weevil adults were associated with the 2020 experimental year. Bees were only found in 2020 early and standard replicates 2 wk after standard application, with none found in the untreated controls (Table 3).

## Discussion

Early application significantly reduced alfalfa weevil populations in our experiment compared to the untreated control, though not always as effectively as the standard application timing. Differences in application timing resulted in shifts in the arthropod community composition in both years, with unique results depending on the year. We observed the potential for early application to reduce negative impacts on beneficial insects – both lady beetles and spiders were more abundant in alfalfa that received early applications than standard applications. Finally, although early application was effective against alfalfa weevil in this experiment, the lack of any scouting protocol to determine whether to use early season applications may result in unnecessary insecticide applications which has many potential implications.

The economic threshold for alfalfa weevil was recently re-examined in Arizona and lowered to 1-3 larvae per sweep given current hay prices and insecticide costs (Harrington et al. 2021). This protocol would typically be employed when alfalfa weevil are present as larvae. At the time of our early applications in both years, alfalfa weevil larvae were rarely found in our plots (Table 2), although we found both adult and egg stages (McClure 2021). At the time of standard application, alfalfa weevil larval abundances were above this economic threshold in both years, so insecticide applications at the site were appropriate given these thresholds (Harrington et al. 2021). We only achieved 78–79% control of alfalfa weevil larvae during both years (Supplemental Table 1) which may suggest pyrethroid resistance development at our site, a possibility given the recent findings of Rodbell et al. (2022) of widespread resistance to pyrethroids in alfalfa weevil, including in Wyoming.

The challenge remains on how to support producers on decision making regarding early applications. We do not know of any scouting guidance based on the adult or egg stages of this pest, therefore early applications are essentially prophylactic. These applications are likely viewed as precautionary – forms of “low-cost insurance” (as described in Ternest et al. 2020). Ternest et al. (2020) worked in Midwestern United States watermelon cropping fields and found that prophylactic applications of pyrethroids targeting striped cucumber beetle were usually unnecessary based on economic thresholds. In our Western alfalfa systems, unnecessary insecticide application likely exacerbates already documented insecticide resistance in alfalfa weevil populations (Rodbell et al. 2022). Insecticide costs will likely increase in the future resulting from both resistance development and the recent loss of chlorpyrifos as an option (Goodhue et al. 2022). Taking these changes into account could further shift economic thresholds. In our region, landscape composition was associated with alfalfa weevil densities (Pellissier et al. 2022). Some growers need treatment every year according to economic thresholds whereas others rarely require treatment. We emphasize the need for future research to explore if scouting protocols based on the adult stage, present earlier in the season, could be an effective strategy to place this practice within an integrated pest management framework. In addition, economic analysis of the impact of early applications would be valuable.

Although very few alfalfa weevil larvae were found in the field at the time of early application in both years, insecticide application at this timing did have efficacy against alfalfa weevil. Pesticide residual efficacy can vary due to various factors including the pesticide’s chemical properties, weather conditions, and plant characteristics (Lyons and Hageman 2021). The main loss process of lambda-cyhalothrin, our applied product in this experiment, is photodegradation. Another study completed at our research site by environmental chemists demonstrated faster loss of lambda-cyhalothrin when morning applied rather than evening applied, since the evening applied pesticide was in darkness for the first 10 h (Kinross 2022). The work of environmental chemists in alfalfa (Lyons and Hageman 2021, Kinross 2022) illustrates that application timing shifts can potentially have pesticide-specific, climate-specific responses via distinct mechanisms. For our experiment, the early application timing in both years occurred at lower air temperatures, higher percent cloud cover, and smaller alfalfa plants than the standard timing (Table 1). The cloudier conditions in early spring may slow rates of loss of insecticide in the field, maintaining efficacy longer to kill emerging alfalfa weevil larvae.

Given negative impacts of insecticide application on beneficials in alfalfa (Michaud and Bain 2016, Prabhaker et al. 2017), we asked if early application could reduce impact on beneficial insects in contrast to insecticide applied later in the season when temperatures have warmed and more beneficial insects are active in alfalfa fields. Our results were mixed and inconsistent across years. Nevertheless, damsel bugs and lady bird beetles were more abundant in plots receiving early treatment than standard treatment in both years, and spiders were more abundant in 1 of the 2 yr. This observation could be due to the timing of emerging insects following overwintering both in the field or nearby, egg laying and emergence of new generations, and response to food sources such as aphids and *Lygus* nymphs. Landscape context is also important to predicting generalist predator densities in alfalfa, potentially more so than prey abundance (Elliott et al. 2002). The early spray timing shows potential for conservation of generalist predators in alfalfa agroecosystems but must be assessed on a larger, production-field scale to account for the possible artifact of small plot work. Our reported differences may be more conservative given movement of insects between experimental plots that would not occur as readily at the production scale. Such findings may also vary in timing if colonization rate by predators differs across large production fields. Finally, the broader landscape context is important to many insects in our study. Alfalfa weevil abundances in a previous Wyoming study were associated with alfalfa and natural habitats in the landscape (Pellissier et al. 2022). Generalist predators in alfalfa in South Dakota were associated with patch diversity, land in the US Department of Agriculture Conservation Reserve Program, wetlands, and woods in the landscape (Elliott et al. 2002). *Lygus* abundances in Arizona were predicted by amount of uncultived habitats, cotton, and seed alfalfa in the landscape (Carrière et al. 2012). Finally, bees in the Midwestern US were positively associated with grasslands, woods, wetlands, and bee-forage crops such as alfalfa (Evans et al. 2018). If one assesses impacts of this practice at a production-field scale, we recommend taking care to design such a study with considerations for broader landscape composition. Our study findings are limited based on only conducting our experiment at 1 research site.

We also were interested in the potential for early spray timing to limit negative impacts on bees. Health and conservation of both honeybees and native bees is a major priority given the importance of bees for pollination and the many threats they face (Belsky and Joshi 2019), and alfalfa is documented to be important to conservation efforts in both the United States and the European Union (Rollin et al. 2013, Evans et al. 2018). Bees were never collected at the time of early application and rarely collected at the time of standard application (Table 2). Two weeks following standard treatment in 2020, we only found bees in the plots that had received insecticide. In 2021, we found no bees in our plots. We hypothesize that both early and standard applications of insecticide reduced pest densities such that alfalfa was able to bloom in 2020, whereas control plots and all of 2021 plots were so defoliated and damaged that no blooms occurred. Integrating conservation and agronomic goals is challenging in this system given the considerable damage caused by alfalfa weevil, and multiple trade-offs to insecticide use and its timing must be examined. These trade-offs were recently detailed in a seed clover system, where insecticide use reduced weevil pest densities, increased crop yield, and profit, but had no negative impacts on weight and reproductive output of bumble bees in colonies (Knapp et al. 2022). In our 2020 experiment, insecticide application, regardless of timing, indirectly impacted bees by protecting the crop so it could reach the bloom stage.

In closing, research on insect pest management in alfalfa must continue to integrate studies of insecticide use with examinations of nontarget effects on beneficial insects given the importance of alfalfa as a conservation-friendly crop. Further detailing trade-offs in distinct pest management scenarios will help to predict potential system and landscape-level impacts of insecticide applications. Identifying these trade-offs can help to best preserve the insect-mediated ecosystem services of both biological pest control and pollination in agricultural landscapes while reducing pest densities so that growers can harvest marketable crops.

## Supplementary Material

Supplementary material is available at *Journal of Economic Entomology* online.

toad071_suppl_Supplementary_Material
