# Supplementary material for: Insecticide application timing effects on alfalfa insect communities
Source: J Econ Entomol. 2023 Apr 21;116(3):815–22. doi: 10.1093/jee/toad071 (PMC10263263; doi:10.1093/jee/toad071)
Supplement: toad071_suppl_Supplementary_Material [file toad071_suppl_supplementary_material.docx]

Supplemental Materials

Supplemental Figure 1. The experimental design is illustrated for A) 2020 and B) 2021. Experimental treatments are indicated with colors: early spray timing (yellow), standard spray timing (orange), and untreated control (green). The experimental plots were embedded within continuously planted, untreated alfalfa (indicated by grey background). To characterize the arthropod community present at the experimental site at the time of treatment, samples were collected from buffer zones throughout the experimental site (indicated in figures as BZ# for distinct samples, data presented in Table 2). All remaining data collection for the experiment occurred within experimental treatment plots to evaluate the impact of our experimental treatments on the response variables of interest (e.g. data presented in Table 3 and Figure 1).

Supplementary Table 1. Arthropod abundance, mean (S.E.), in early application, standard application, and control treatments per 20 sweeps. These collections occurred roughly one week after the standard application data in each year.

|  | June 4, 2020 | | |  | June 1, 2021 | | |
| --- | --- | --- | --- | --- | --- | --- | --- |
|  | Early | Standard | Control |  | Early | Standard | Control |
| **Alfalfa weevil (larvae)** | 186 (41) | 172 (35) | 768.2 (212) |  | 109.6 (13) | 66.8 (12) | 316.6 (36.6) |
| **Aphids** | 394 (112) | 298 (82) | 135.2 (53) |  | 445.4 (26) | 172.8 (7.8) | 878 (49) |
| **Grasshoppers** | 24 (6.35) | 1.8 (0.9) | 28.4 (4) |  | 0.2 (0.2) | 0 | 1 (0.3) |
| ***Lygus* bugs** | 8 (1.8) | 1.6 (0.2) | 51.6 (11) |  | 0 | 0 | 31.6 (13) |
| **Damsel bugs** | 3.2 (0.6) | 0 | 4.2 (1.7) |  | 0.2 (0.2) | 0 | 2 (0.5) |
| **Wasps** | 5.4 (1.8) | 4 (1.5) | 4.6 (1.2) |  | 7.6 (0.2) | 4.4 (0.7) | 12.2 (1.2) |
| **Ladybird beetles** | 4.6 (1.4) | 1.9 (0.8) | 4 (0.9) |  | 0.2 (0.1) | 0 | 0.4 (0.2) |
| **Spiders** | 0.4 (0.4) | 1.4 (0.4) | 3.6 (1.0) |  | 0.4 (0.5) | 1.8 (0.8) | 1.1 (0.2) |
| **Bees** | 0.8 (0.2) | 0.6 (0.2) | 0.4 (0.2) |  | 0 | 0 | 0 |
|  |  |  |  |  |  |  |  |
